# UV‐A Radiation Impairs Sebaceous‐Gland‐Related Skin Barrier Function by Inducing Inflammation and Altering Intracellular Sebum‐Like Lipid Composition

**DOI:** 10.1111/jocd.70392

**Published:** 2025-10-10

**Authors:** Wangyue Dong, Maori Kono, Masayuki Takaishi, Ayumi Kyuka, Hiroko Kato, Fumitaka Fujita

**Affiliations:** ^1^ Laboratory of Advanced Cosmetic Science, Graduate School of Pharmaceutical Sciences Osaka University Suita Japan; ^2^ Advanced Technology Institute Mandom Corporation Chuo‐ku Japan

**Keywords:** inflammation, sebaceous glands, sebocytes, sebum, skin barrier, UV‐A

## Abstract

**Background:**

Sebaceous glands (SGs) secrete sebum to form a protective barrier that maintains skin health. However, exposure to ultraviolet A (UV‐A) radiation damages the skin barrier, leading to dryness and inflammation.

**Aims:**

In this study, we investigated how UV‐A radiation alters SG function, focusing on inflammation and changes in the composition of intracellular sebum‐like lipids. We also explored the role of SG‐related mechanisms in UVA‐radiation‐induced skin barrier damage.

**Methods:**

Human primary sebocytes were cultured from SGs isolated from human skin samples and exposed to different doses of UV‐A radiation. Inflammatory cytokines released into the culture medium were measured, followed by gene expression analysis using mRNA extracted from cells to examine specific target genes. Intracellular sebum‐like lipid composition was analyzed using liquid chromatography–tandem mass spectrometry, and skin barrier function was evaluated using a three‐dimensional reconstructed human epidermis (3D skin) model.

**Results:**

UV‐A irradiation increased inflammatory cytokine levels in the culture medium and altered the expression levels of several genes. Intracellular sebum‐like lipid composition was also modified following UV‐A irradiation, with notable sex differences. Furthermore, the skin barrier function was impaired in 3D skin models cultured with the supernatant from UV‐A‐irradiated sebocyte cultures.

**Conclusions:**

This study demonstrated that UV‐A radiation stimulated sebocytes to release inflammatory cytokines and altered gene expression. Additionally, UV‐A irradiation modified the intracellular sebum‐like lipid composition in a sex‐dependent manner, contributing to skin barrier damage.

## 1 Introduction

The sebaceous gland (SG) is a skin organ located in the dermis across almost the entire human body, except for the palms and soles. Its primary function is to secrete sebum via a unique mechanism known as holocrine secretion, in which sebocytes mature, break down, and release their contents as sebum onto the skin surface. Sebum is composed of various lipids, including triglycerides (TGs), wax esters, and squalene, and it plays a crucial role in forming a protective hydrophobic layer that prevents moisture loss and shields the skin from external stimuli, such as viruses and pollutants [1]. Because sebum is essential for maintaining skin homeostasis, any dysfunction in this process can lead to skin disorders. Excessive sebum production and alterations in its composition have been linked to skin inflammation [1, 2, 3], while SG atrophy and sebum deficiency are associated with increased moisture loss and compromised skin barrier function [2].

Among the factors that regulate SG function, internal factors, such as hormones, age, and menstruation, are well understood. However, the effect of external factors, particularly ultraviolet (UV) radiation, remains poorly understood. The UV rays that reach the Earth's surface consist of approximately 95% long‐wavelength UV‐A radiation, which penetrates deep into the dermis where SGs reside. Previous studies have shown that UV‐A oxidizes TGs, a major component of sebum, causing dry skin, damaging the skin barrier, and triggering inflammation [4]. However, the precise mechanism underlying these effects remains unclear.

To better understand SG function and its response to environmental factors, such as UV‐A radiation, a reliable in vitro model is essential. According to Marlon et al., several immortalized sebocyte cell lines, such as SZ95 and SEB‐1, have been developed and are widely used in research [5]. However, some of these cell lines lack the key characteristics of primary human sebocytes, limiting their applicability in certain studies [5], especially in understanding the correlation with age or sex. Unlike immortalized cell lines, primary sebocytes retain essential physiological properties, including the ability to produce sebum, making them valuable models for studying changes in gene expression and lipogenesis‐related mechanisms [5]. Therefore, we first developed an improved method for culturing primary human sebocytes based on previously established techniques [6]. Overall, our study aimed to uncover the underlying mechanisms by which UV‐A irradiation influences intracellular accumulation of sebum‐like lipid and skin barrier function, providing new insights into potential treatments for SG‐related diseases.

## 2 Materials and Methods

### 2.1 Human Skin Materials

All human skin samples used in this study were surplus materials from plastic surgeries, provided by Seishin Plastic and Aesthetic Surgery Clinic Co. Ltd. (Tokyo, Japan). All studies were performed in accordance with relevant guidelines and regulations. Informed consent was obtained from all the participants. Skin samples from male and female individuals aged 32–60 years were used in this study.

### 2.2 Isolation of Sebaceous Glands

Human skin tissues cut into small squares were used for the isolation of SGs. Before SG isolation, fat and subcutaneous tissue were carefully removed, and the skin tissues were cut into 3 mm squares. For enzyme treatment, skin pieces were placed with the epidermis facing upward in 60 mm dishes containing 5 mL of 20 U/mg dispase. The skin pieces were incubated in this solution at 4°C overnight. After incubation, the epidermis was gently separated from the dermis to expose the SGs attached to the epidermis. The SGs were then collected using scissors and tweezers.

### 2.3 Primary Human Sebocyte Culture

Isolated SGs were placed in six‐well plates (Falcon; Corning Inc., Corning, NY, USA) coated with 7% Matrigel (Corning Inc.), with approximately 3–5 SGs per well. The culture medium consisted of Dulbecco's modified Eagle medium (DMEM)/Ham's F‐12 with l‐glutamine and phenol red (Wako Pure Chemical Corporation, Osaka, Japan), supplemented with 10% fetal bovine serum (FBS; Thermo Fisher Scientific, Waltham, MA, USA), 1% penicillin–streptomycin‐amphotericin B suspension (AA; Wako), 5 ng/mL human recombinant EGF protein (Gibco), and 5 μM CultureSure Y‐27632 (Fujifilm Corporation, Tokyo, Japan). To ensure that the SGs adhered to the bottom of the wells rather than floating, a minimal amount of medium was initially added when seeding them. This approach, known as the air‐liquid interface culture method, facilitates efficient attachment. The SGs were allowed to attach overnight before the medium was replaced with 1 mL of the same formulation. After approximately 3 weeks of culture, primary human sebocytes were harvested by using Trypsin–EDTA (0.5%), no phenol red (Gibco) for subsequent experiments.

### 2.4 Immunocytochemistry

Primary human sebocytes cultured in the Nunc Lab‐Tek II Chamber Slide System (8‐well) (Thermo Fisher Scientific) were fixed with 4% paraformaldehyde and washed with Dulbecco's phosphate‐buffered saline (PBS) (−). Fixed sebocytes were blocked with 5% bovine serum albumin in PBS with 0.1% Triton X‐100 (PBST) at 4°C for at least 30 min. An anti‐mouse keratin‐7 antibody (GeneTex, Irvine, CA, USA; 1:200) was used as the primary antibody, and anti‐mouse IgG (Thermo Fisher Scientific, 1:500) served as the secondary antibody. The nuclei were stained with Hoechst 33342 (Thermo Fisher Scientific, 1:1000). After a 2‐h incubation at room temperature in the dark, the sebocytes were washed three times with PBST, then mounted with FluorSave reagent (Sigma‐Aldrich, Merck KGaA, Darmstadt, Germany) and covered with a cover glass for observation using a confocal laser‐scanning microscope (FV3000; Olympus Corporation, Tokyo, Japan).

### 2.5 Cell Culture and Three‐Dimensional Skin Culture

Primary human sebocytes obtained from CTI Biotech (Lot: SK‐0390, Caucasian, 57‐year‐old female; CTI Biotech, Lyon, France) were cultured in Sebomed basal medium (Sigma‐Aldrich) supplemented with 10% FBS and 1% AA. The cells were grown in a 75 cm^2^ rectangular canted neck cell culture flask (Corning) coated with fibronectin under conditions of 37°C, 5% CO_2_, and were used at passage 6 (P6) at the time of the experiment. Normal human dermal fibroblasts (NHDFs; Catalog No. KF‐4009; KURABO Industries Ltd., Osaka, Japan) were cultured in high‐glucose DMEM (Sigma‐Aldrich) supplemented with 5% FBS and 1% AA under conditions of 37°C, 5% CO_2_, and were also used at P6 in this study. A three‐dimensional reconstructed human epidermis (3D skin) model, LabCyte EPI‐MODEL 24, composed of normal human epidermal keratinocytes forming a multilayered structure (Japan Tissue Engineering), was maintained according to the manufacturer's instruction manuals under conditions of 37°C and 5% CO_2_.

### 2.6 Lipogenesis Assay

Sebocytes cultured in six‐well plates were treated with high‐glucose DMEM without l‐glutamine and phenol red (Wako), with 10^−3^ M linoleic acid (Sigma‐Aldrich) diluted in ethanol (Wako) and 1 μg/mL Nile Red (Wako). The medium was changed every day for 3 days, followed by a 3‐day rest period. Fluorescent images were captured approximately 1 week after UV‐A exposure using an all‐in‐one fluorescence microscope (BZ‐X710; Keyence Corporation, Osaka, Japan).

### 2.7 UV‐A Irradiation

UV‐A irradiation was performed using UV bench lamps (UVP, Model 95‐0042‐07, 365 nm; Analytik Jena GmbH, Jena, Germany) at an intensity of 1.7 mW/cm^2^. Before irradiation, the culture medium was replaced with 2 mL of DMEM/F‐12 without phenol red (Gibco). Sebocytes were exposed to 0, 0.5, 1, and 5 J/cm^2^ UV‐A irradiation by adjusting the duration.

### 2.8 Inflammation Mediator Detection

Sebocyte culture supernatants were collected 48 h after UV‐A irradiation to measure inflammatory mediators. For each sample, 50 μL of supernatant was analyzed using the ProcartaPlex Multiplex Assay Kit (Thermo Fisher Scientific) according to the manufacturer's protocol. Eighteen cytokines, including interleukin (IL)‐13, IL‐17a, IL‐1α, IL‐1β, IL‐1 receptor antagonist (IL‐1RA), IL‐2, IL‐23, IL‐31, IL‐33, IL‐4, IL‐5, IL‐6, IL‐7, and IL‐8, tumor necrosis factor‐alpha, tumor necrosis factor‐beta, thymic stromal lymphopoietin, and vascular endothelial growth factor‐D were detected using the BioPlex200 system (Bio‐Rad Laboratories, Hercules, CA, USA).

### 2.9 RNA Experiments

Cells were collected 48 h after UV‐A irradiation. Total RNA was extracted using a NucleoSpin Plus XS kit (Macherey‐Nagel GmbH & Co. KG, Düren, Germany). For DNA microarray analysis, 250 ng of total RNA was processed using the Clariom S Array, human (Thermo Fisher Scientific), following the manufacturer's protocol. For reverse transcription‐quantitative polymerase chain reaction (RT‐qPCR), total RNA was reverse‐transcribed into cDNA using SuperScript IV VILO Master Mix (Thermo Fisher Scientific), and TaqMan Universal Master Mix II, no UNG (Thermo Fisher Scientific), was used for qPCR reactions. Specific TaqMan probes were used to determine gene expression levels (Table S1).

### 2.10 Lipid Extraction and Quantification

Linoleic acid was added to the culture medium to induce lipid synthesis in sebocytes that had reached sufficient confluency, both in the non‐treated groups and in those exposed to UV‐A, 48 h after irradiation. After continuous culture for one week, lipids were extracted using the Folch method, and the resulting solution was dried under a stream of nitrogen gas. The dried lipid samples were dissolved in 1 mL of hexane (Wako) and sonicated for 5 min. The supernatant was transferred to a liquid chromatography (LC)–mass spectrometry (MS) autosampler vial (Waters Corporation, Milford, MA, USA) and diluted 20‐fold with 2‐propanol (Wako). Lipid analysis was performed using an LC–MS system (Waters, Acquity UPLC H‐class) equipped with an Acquity UPLC BEH C8 column. Tristearin (Wako) was used as the standard, and MassLynx (Waters) was used for calculations and data analysis.

### 2.11 Trans‐Epidermal Water Loss (TEWL) Measurement

Conditioned media from SGs were collected 48 h after UV‐A exposure and stored at −80°C until use. For TEWL analysis, 3D skin models were cultured with conditioned media derived from SGs at a 100‐fold dilution (1%) in the applied culture medium for 48 h prior to TEWL measurements. The skin models were equilibrated at room temperature under atmospheric conditions for 30 min. TEWL on the epidermal surface was measured in duplicate using an Evaporimeter AS‐TW1 (ASAHI BIOMED, Yokohama, Japan). Each measurement was performed for approximately 60 s or until a stable reading was obtained, as determined using the device software. The measurements were performed under sterile conditions.

### 2.12 Statistical Analysis

All statistical analyses were performed using GraphPad Prism software (version 9.5.1 [733], GraphPad Software, San Diego, CA, USA). One‐way analysis of variance was used to compare differences among groups, followed by Tukey's multiple‐comparisons test for post hoc analysis. Data are presented as the mean ± standard deviation. Statistical significance was set at *p* < 0.05.

## 3 Results

### 3.1 Isolation of Human SGs and Culture of Primary Human Sebocytes

**FIGURE 1:**
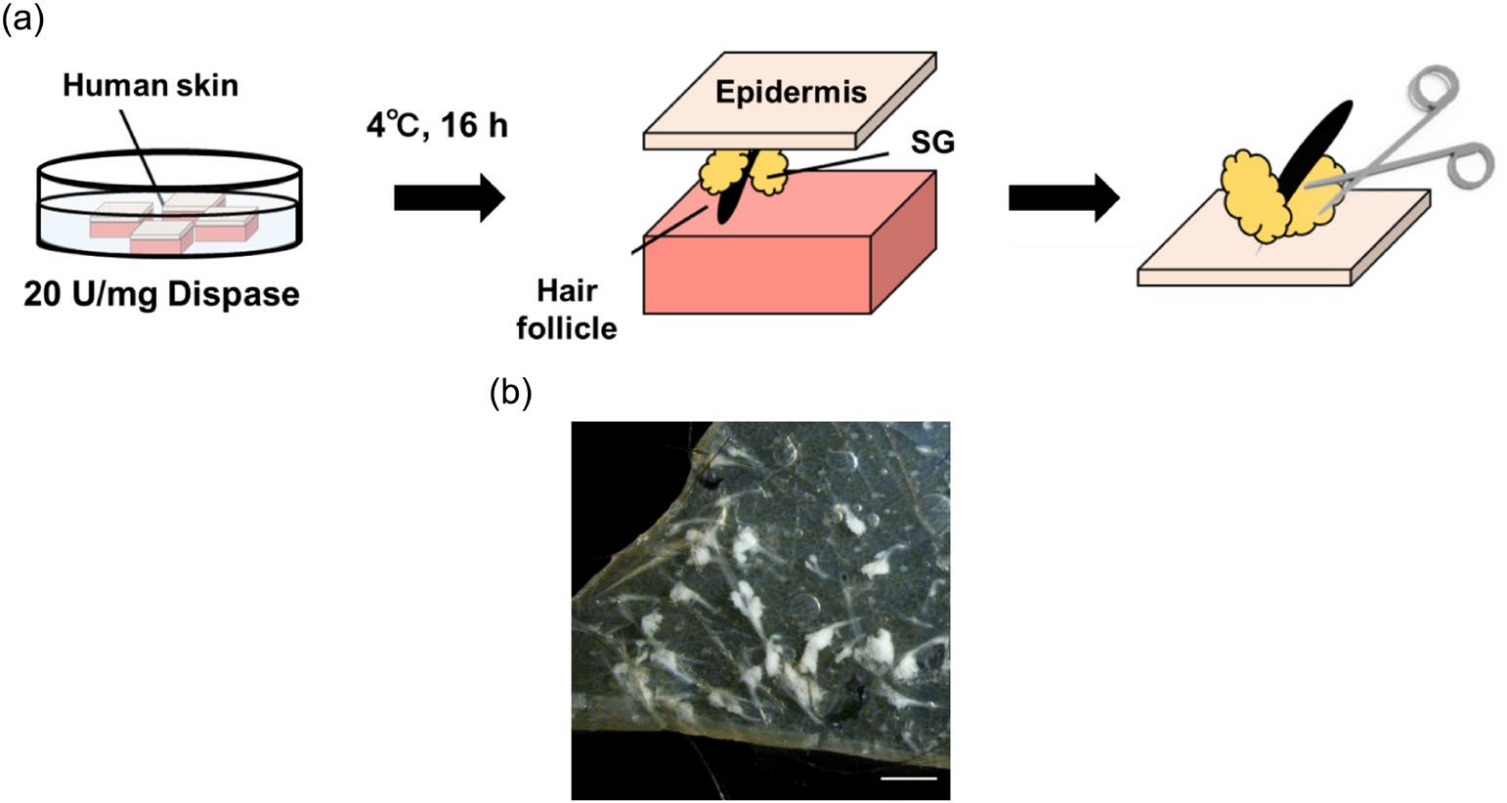
Isolation of sebaceous glands (SGs) from human skin. (a) SGs were isolated from human skin treated with dispase overnight. (b) SGs attaching to the epidermis. Scale bar: 100 μM.

To investigate how UV‐A irradiation influences the sebum production function of SGs, sebocytes were cultured from isolated human skin. Following a previously established method [6], the skin was treated with dispase overnight before SGs were isolated from the peeled‐off epidermis (Figure 1a,b).

**FIGURE 2:**
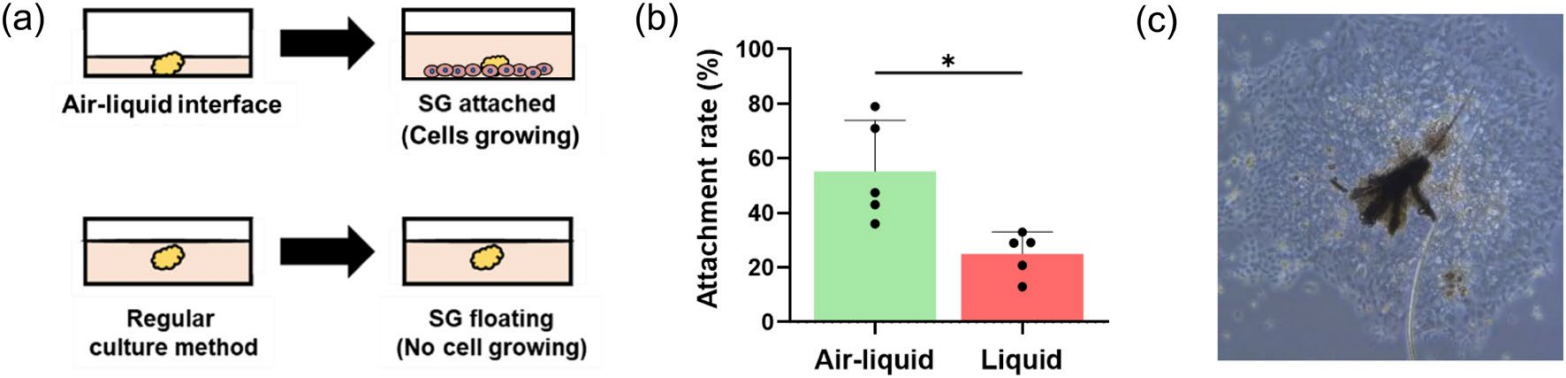
Primary human sebocytes cultured with isolated SGs. (a) SGs cultured using the air‐liquid interface method. (b) SGs seeded with the air‐liquid interface culture method show a higher attachment rate (*n* = 5). *p < 0.05. (c) Sebocytes are growing out from SGs.

Freshly isolated SGs were seeded onto plates pre‐coated with Matrigel using the air‐liquid interface culture method (Figure 2a), which significantly enhanced the attachment rate of the SGs (Figure 2b). Once attached, the sebocytes began to grow out from the explant within approximately 3 days (Figure 2c).

### 3.2 Characterization of Cultured Primary Human Sebocytes

To characterize the cultured primary human sebocytes, their gene expression profile was compared with that of commercially available sebocytes and NHDFs (Figure S1a–f). Expression levels of the early sebocyte differentiation markers Mucin 1 (*MUC1*) and PR/SET Domain 1 (*PRDM1*) were low in cultured sebocytes and even lower than those observed in NHDFs (Figure S1a,b). In contrast, the sebocyte‐associated gene Melanocortin 5 Receptor (*MC5R*) was specifically detected in cultured sebocytes but was undetectable in NHDFs, although its overall expression level remained relatively low (Figure S1c). Notably, the lipid synthesis‐related enzymes Fatty Acid Synthase (*FASN*) and Stearoyl‐CoA Desaturase 1 (*SCD*) were expressed in cultured sebocytes but were absent or expressed at negligible levels in NHDFs (Figure S1d,e), indicating partial retention of sebaceous metabolic function. Furthermore, the expression of Keratin 7 (*KRT7*), a marker associated with sebocyte cytoskeletal differentiation, was elevated in cultured sebocytes compared to both commercially available sebocytes and NHDFs (Figure S1f).

**FIGURE 3:**
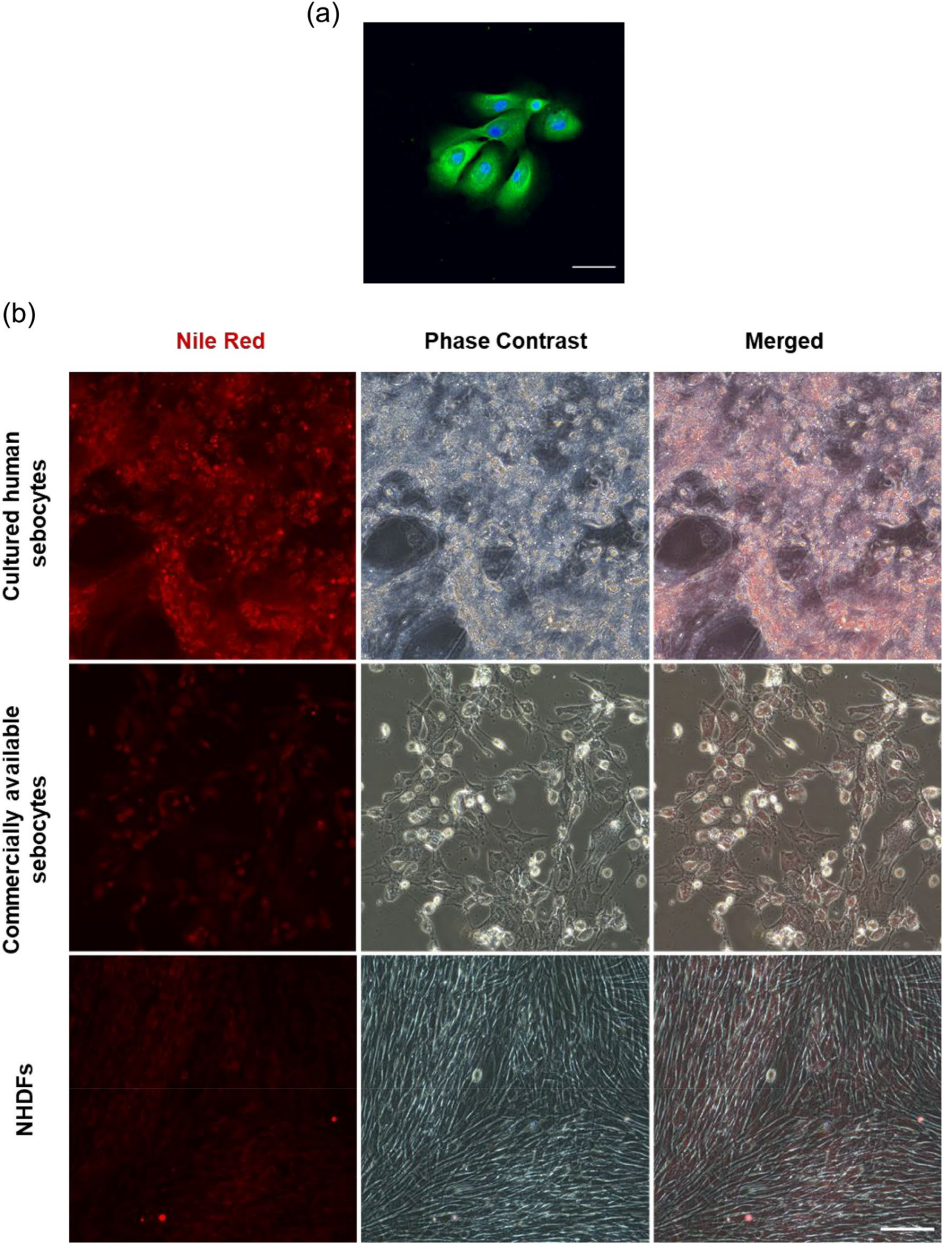
Characterization of cultured human sebocytes compared with commercially available sebocytes and NHDFs. (a) Cultured sebocytes are positive for *KRT7*. Scale bar: 50 μm. (b) Cultured sebocytes produce oil droplets when stimulated with linoleic acid, similar to commercially available sebocytes, but different from normal human dermal fibroblasts (NHDFs). Scale bar: 100 μm.

To further define the characteristics of the cultured primary human sebocytes, immunocytochemistry was performed, which confirmed that they were *KRT7*‐positive (Figure 3a). Additionally, a lipogenesis assay was performed to assess the ability of sebocytes to produce intracellular sebum‐like lipids. Cultured sebocytes demonstrated the ability to accumulate intracellular sebum‐like oil droplets comparable to those produced by commercially available sebocytes, whereas no sebum‐like oil droplets were observed in NHDFs (Figure 3b). These findings indicated that primary sebocytes cultured using our method retained their characteristic features and functional ability to accumulate sebum‐like lipids.

Moreover, RT‐qPCR analysis revealed that cultured primary human sebocytes exhibited expression of several genes specifically involved in lipid biosynthesis compared to commercially available sebocytes (Table 1). While both cell types showed comparable expression levels of genes such as Farnesyl‐diphosphate farnesyltransferase 1 (*FDFT1*) and Sterol‐C5‐desaturase (*SC5D*), markedly higher expression levels were observed in cultured sebocytes for genes including 7‐dehydrocholesterol reductase (*DHCR7*), *MC5R*, Acetyl‐CoA carboxylase alpha (*ACACA*), and Fatty acyl‐CoA reductase 1 (*FAR1*).

**TABLE 1:** Semi‐quantitative RT‐qPCR analysis of lipid metabolism–related genes.

Gene symbol	Cultured human sebocytes	Commercially available sebocytes
*FDFT1*	+++	+++
*SC5D*	+++	+++
*SREBF1*	+++	++
*FASN*	+++	++
*SCD*	+++	++
*HMGCS1*	+++	++
*HMGCR*	+++	++
*SQLE*	+++	++
*DHCR24*	+++	++
*DHCR7*	+++	+
*SCD5*	++	++
*MC5R*	+	−
*ACACA*	+	−
*FAR1*	+	−

*Note:* The gene expression level of related genes as they are scored relative to ACTB using the following semi‐quantitative scale: “−” for values ≤ 0.0001, “+” for 0.0001–0.001, “++” for 0.001–0.01, and “+++” for values > 0.01.

Abbreviations: *DHCR24*, 24‐dehydrocholesterol reductase; *HMGCR*, 3‐hydroxy‐3‐methylglutaryl‐CoA reductase; *HMGCS1*, 3‐hydroxy‐3‐methylglutaryl‐CoA synthase 1; *SCD5*, stearoyl‐CoA desaturase 5; *SQLE*, squalene epoxidase; *SREBF1*, sterol regulatory element‐binding transcription factor 1.

### 3.3 Influence of UV‐A Irradiation on the Inflammatory Response of Sebocytes

**FIGURE 4:**
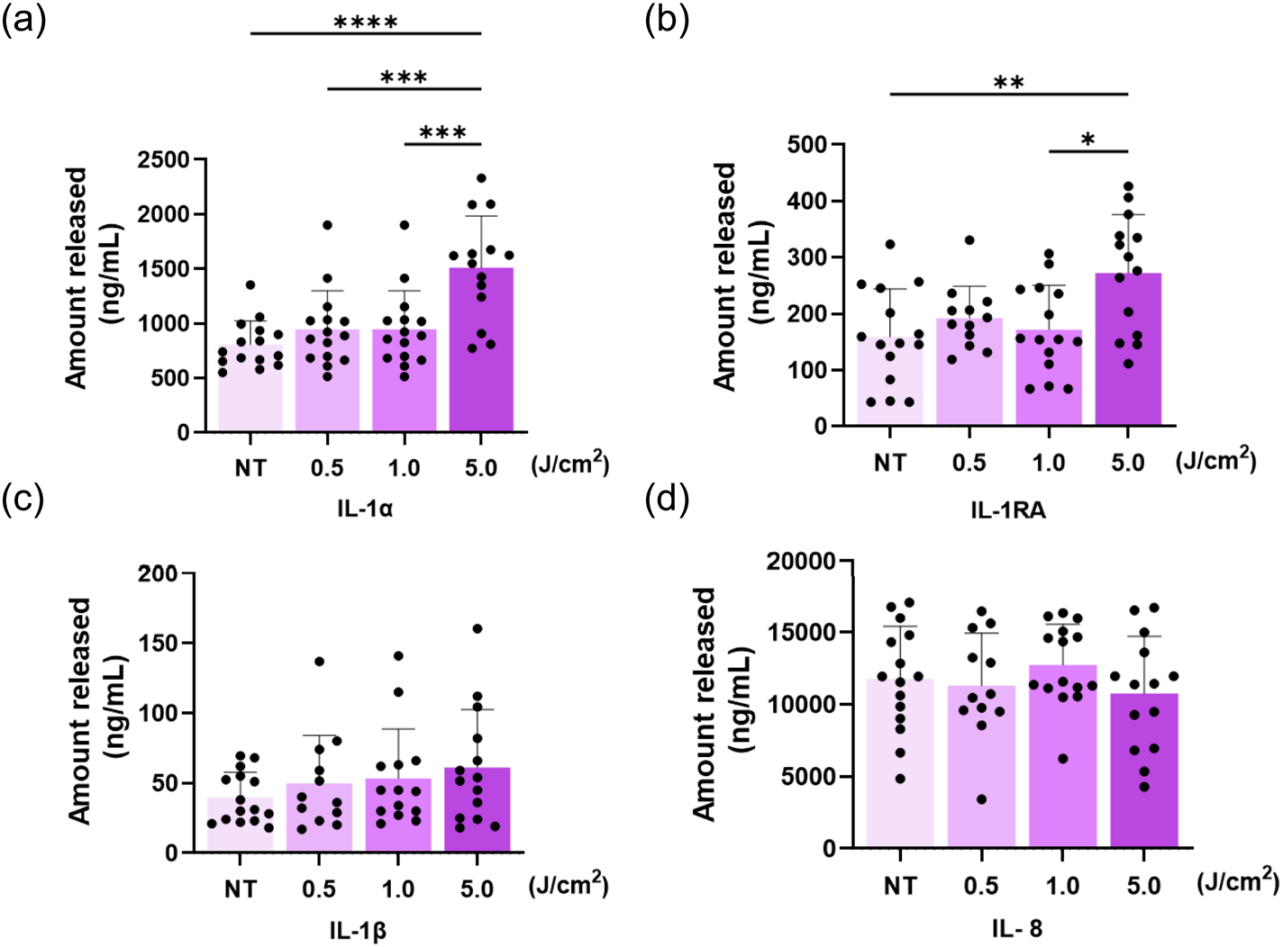
Inflammatory cytokines released from sebocytes into the cell culture supernatant. (a, b) IL‐1α and IL‐1RA in the cell culture supernatant increased dose‐dependently following UV‐A irradiation. (c) IL‐1β in the cell culture supernatant showed a tendency to increase. (d) IL‐8 exhibited the highest release among all detected cytokines, yet no significant change was induced by UV‐A (*n* = 12–15). *p < 0.05, **p < 0.01, ***p < 0.001, ****p < 0.0001.

To investigate how UV‐A irradiation influences SGs, the effect of UV‐A exposure on inflammatory cytokine release from sebocytes was evaluated. To treat the sebocytes, the UV‐A radiation doses were at intervals between 0 and 5 J/cm^2^. Inflammatory cytokine levels were measured in the culture supernatants 48 h after UV‐A irradiation. The results showed that the levels of IL‐1α and IL‐1RA in the supernatant increased in a dose‐dependent manner following treatment (Figure 4a,b). IL‐1β also exhibited a slight trend of increased levels in response to UV‐A irradiation (Figure 4c), while IL‐8 showed no changes between groups, but remained the most abundantly released cytokine among those detected (Figure 4d). On the other hand, under identical conditions, NHDFs released significantly lower levels of same cytokines compared to sebocytes, with little to no change across increasing UV‐A does groups (Figure 2a–c). In contrast, IL‐8 levels were consistently higher in NHDFs than in sebocytes across all UV‐A dose groups, yet the overall release remained unchanged with increasing irradiation (Figure 2d).

### 3.4 Altered Gene Expression Levels in Sebocytes Induced by UV‐A Irradiation

**FIGURE 5:**
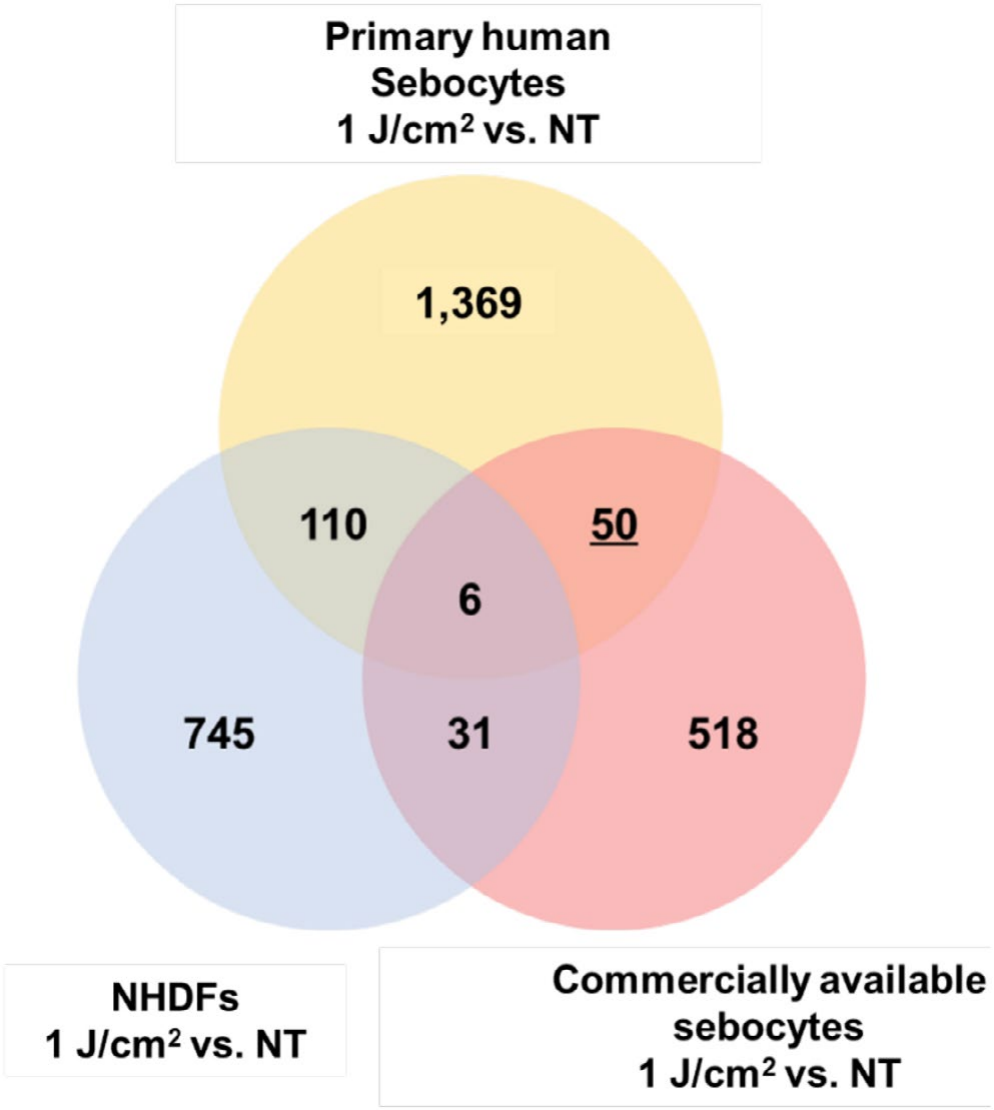
DNA microarray results showing changes in gene expression levels in cultured sebocytes induced by UV‐A irradiation. DNA microarray analysis revealed 50 genes with altered expression levels specifically in cultured sebocytes after UV‐A irradiation compared to their levels in the NT group (*n* = 2).

To investigate the underlying causes of increased inflammatory cytokine release by sebocytes, DNA microarray analysis was performed to identify gene expression changes in sebocytes treated with 1 J/cm^2^ UV‐A irradiation compared to the non‐treated group (the NT group). By comparing the results for sebocytes with those for commercially available sebocytes and NHDFs, this analysis identified 50 genes that exhibited specific changes in expression levels in cultured sebocytes (Figure 5).

**FIGURE 6:**
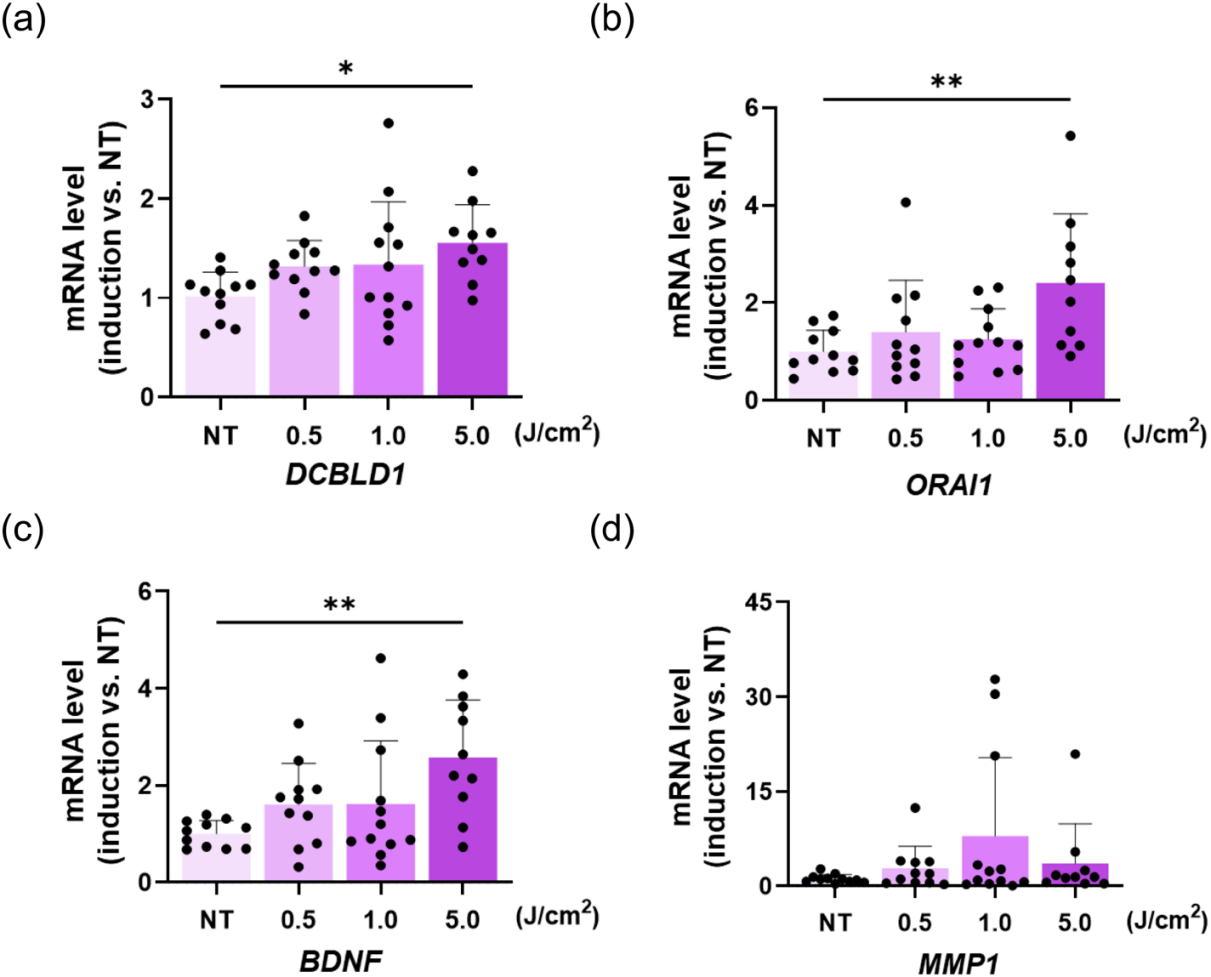
From the DNA microarray results, 26 genes were selected for validation using reverse transcription‐quantitative polymerase chain reaction. (a–c) Expression of the genes encoding *DCBLD1*, *ORAI1*, and *BDNF* increased in a dose‐dependent manner after UV‐A irradiation. (d) *MMP1* levels showed a tendency to increase (*n* = 10–12). *p < 0.05, **p < 0.01.

Among these 50 genes, 26 genes with significant changes and functional relevance were selected for further validation using RT‐qPCR. The results revealed that the expression levels of genes encoding discoidin, CUB, and LCCL‐domain‐containing 1 (*DCBLD1*), calcium release‐activated calcium channel protein 1, *ORAI1*, and brain‐derived neurotrophic factor (*BDNF*) increased in a dose‐dependent manner following UV‐A irradiation (Figure 6a–c). Additionally, matrix metallopeptidase/metalloproteinase 1 (*MMP1*) showed an increasing trend, although there was no statistically significant change (Figure 6d).

### 3.5 Influence of UV‐A Irradiation on the Intracellular Sebum‐Like Lipid Composition of Sebocytes

**FIGURE 7:**
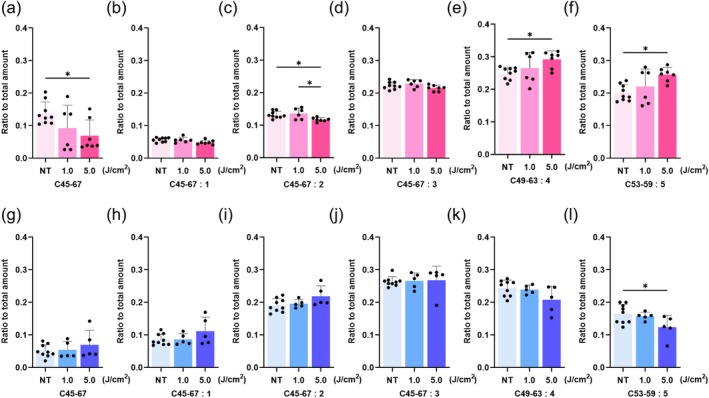
UV‐A irradiation alters the composition of triglycerides (TGs) in sebocytes. (a–f) In TGs extracted from female‐derived sebocytes, the ratio of the levels of saturated and unsaturated TGs with two double bonds (Carbon, “C”; C45‐57:2) decreased, while the levels of unsaturated TGs with four or five double bonds (C49‐63:4, C53‐59:5) increased after UV‐A irradiation. (g–l) In TGs extracted from male‐derived sebocytes, the ratio of the levels of unsaturated TGs with five double bonds (C53‐59:5) decreased after UV‐A irradiation (*n* = 5–9). (a, b) Saturated TGs. (b–f, h–l) Unsaturated TGs. *p < 0.05.

Next, to evaluate the effect of UV‐A irradiation on intracellular sebum‐like lipid composition, changes in the intracellular profile of TGs were analyzed following UV‐A exposure. Linoleic acid was added to sebocytes 48 h after UV‐A irradiation to promote lipid synthesis without interfering with UV‐A–induced cellular responses. Sebocytes were then collected one week later for lipid extraction. The lipid composition of TGs with carbon chain length 45 to 67 (C45‐67) was analyzed and quantified using LC–MS/MS. These results revealed sex‐dependent differences in sebocyte responses to UV‐A irradiation. In female‐derived sebocytes, the ratio of the levels of saturated and unsaturated TGs with two double bonds decreased, while the levels of unsaturated TGs with four and five double bonds increased relative to the total TG content after UV‐A irradiation (Figure 7a–f). In contrast, in male‐derived sebocytes, the ratio of the levels of unsaturated TGs with five double bonds decreased, whereas other TGs showed minimal changes in response to UV‐A irradiation (Figure 7g–l).

### 3.6 Influence UV‐A Irradiated Sebocyte Culture Supernatant on Skin Barrier Function in a 3D Cultured Skin Model

To elucidate the effects of UV‐A irradiation‐induced sebocyte secretions on skin barrier function, a 3D cultured skin model was treated with sebocyte culture supernatant, and TEWL, a critical parameter of skin barrier integrity, was evaluated. The expression levels of genes associated with skin barrier function were quantified using RT‐qPCR.

**FIGURE 8:**
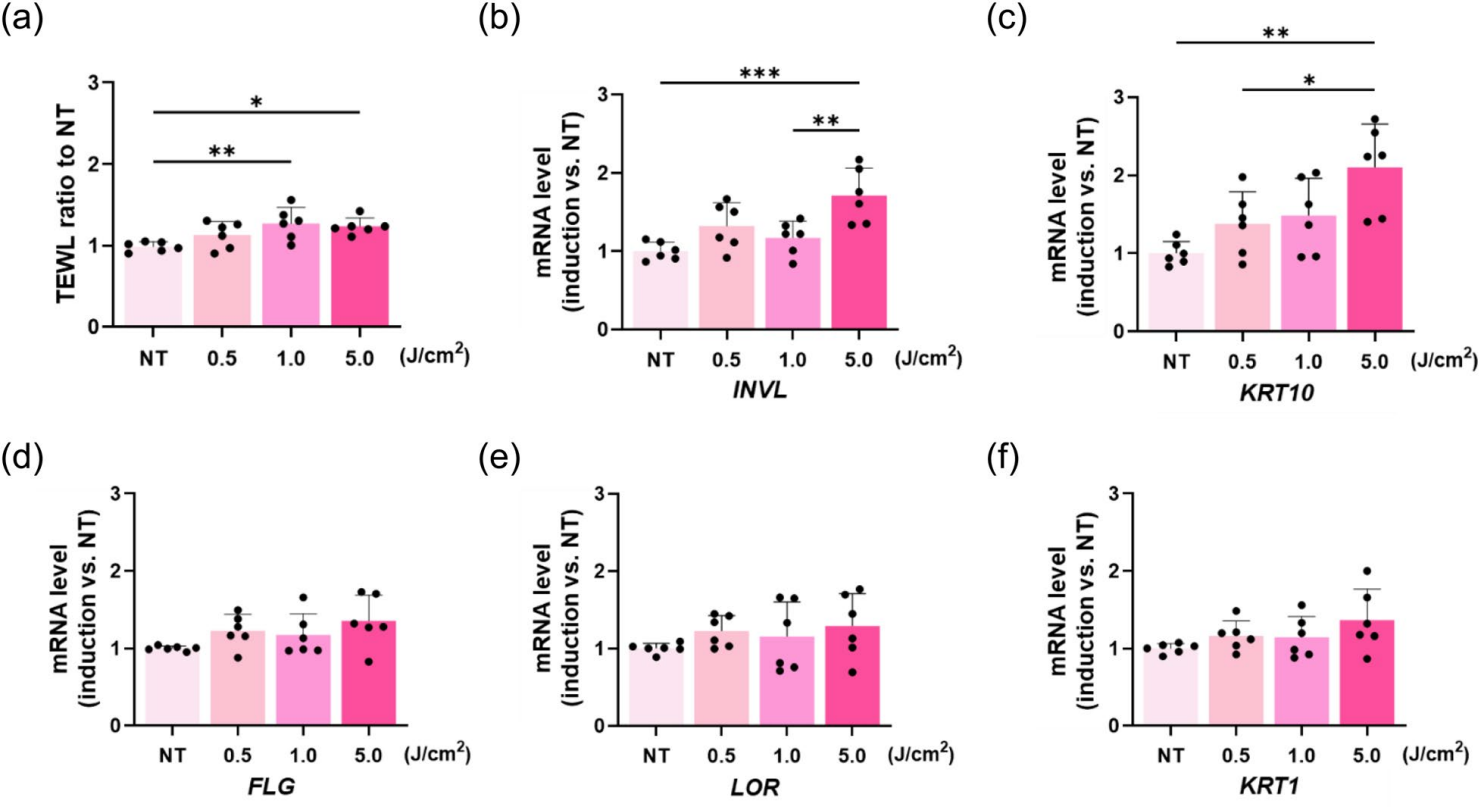
Analysis of skin barrier function–related factors. (a) Culture supernatant from UV‐A–irradiated sebocytes led to a higher TEWL ratio in the 3D cultured skin model. (b, c) *IVNL* and *KRT10* expression levels increased in a dose‐dependent manner in the 3D skin model cultured with sebocyte supernatant. (d–f) *FLG*, *LOR*, and *KRT1* levels showed a similar trend, but the changes were not statistically significant. (*n* = 6). *p < 0.05, **p < 0.01, ***p < 0.001.

The results indicated that treatment with UV‐A irradiation stimulated the sebocyte culture supernatant, leading to a significant increase in the TEWL ratio in the 3D skin model (Figure 8a). RT‐qPCR analysis further demonstrated the dose‐dependent upregulation of involucrin (*INVL*) and keratin 10 (*KRT10*) gene expression levels in response to UV‐A‐irradiated supernatant from sebocyte culture (Figure 8b,c). Although similar trends were observed for filaggrin (*FLG*), loricrin (*LOR*), and keratin 1 (*KRT1*), these changes were not statistically significant (Figure 8d–f).

## 4 Discussion

In this study, we demonstrated an efficient method for culturing primary human sebocytes using an air‐liquid interface culture method to seed SGs isolated from human skin. Previous studies have attempted to culture primary human sebocytes using various protocols that focused on improving SG attachment to culture dishes [6]. To enhance the sebocyte growth efficiency, we used the air‐liquid interface culture method on the first day of seeding, which significantly improved attachment without causing gland dehydration or complicating the procedure. Using our technique, sebocytes were efficiently isolated without contamination by other cell types. Functional tests confirmed the cultured sebocytes' ability to accumulate sebum‐like oil droplets in vitro.

As previously reported, primary sebocytes are essential for marker identification and pathway analysis; however, they proliferate at a relatively slow rate [5], a finding that was confirmed in our study. To obtain a sufficient sebocyte population, it is advisable to seed a larger number of SGs while optimizing attachment efficiency. Typically, sebocytes reach a satisfactory population after < 2 weeks, although growth rates may vary depending on the donor, as SG characteristics are influenced by multiple factors [7, 8]. Although primary sebocyte cultures can be maintained for more than 1 month, fibroblast overgrowth has become a concern because of the slow proliferation rate of sebocytes [5] and their holocrine secretion characteristics.

Several immortalized sebocyte cell lines, including SZ95 and SEB‐1, have been developed to overcome the limitations of primary cultures. These cell lines have enhanced proliferative potential but may suffer from limited biological diversity, as they are mostly derived from single donors, whereas sebaceous gland characteristics vary significantly by sex, age, and anatomical location [7, 8, 9, 10]. Additionally, some immortalized sebocytes express only a limited number of SG markers [5, 9, 10]. In contrast, we used human skin samples from both male and female donors across a broader age range (30 to 60s), providing a more representative model of sebocyte biology. These efforts to collect and culture primary human sebocytes are essential for advancing future research on SG function and related mechanisms.

The differentiation state of our cultured primary human sebocytes was evaluated by examining the expression of key sebocyte‐associated markers. We found that early differentiation markers such as *MUC1* and *PRDM1* were expressed at low levels. In contrast, *MC5R* was specifically detected in the cultured sebocytes, indicating lineage specificity. The low expression of *PRDM1* suggests that these cells may represent an intermediate stage of sebocyte differentiation. This idea is further supported by the expression of *FASN* and *SCD*, two lipogenesis‐associated enzymes [11], indicating the initiation of lipid accumulation. This combination of early lipid synthesis and absent terminal markers such as *PPARG* supports the interpretation that the sebocytes are in an intermediate stage of differentiation. We also observed a strong expression of *KRT7*. Although *KRT7* is not sebocyte‐specific, it is widely used as a marker for sebocytes [12], and its robust expression in our cultures supports sebocyte lineage identity at this differentiation stage.

When we compared our primary sebocytes with commercially available sebocytes, we found that while both the cultured human primary sebocytes and commercially available sebocytes were capable of producing intracellular sebum‐like lipids, they differ in several aspects. Both cell types exhibited similar expressions of certain genes such as *FDFT1* and *SC5D*, but showed notable differences in the expression of *DHCR7*, *MC5R*, *ACACA*, and *FAR1*. Taken together with results from SG marker comparisons and a broader DNA microarray dataset (data not shown), these findings suggest that although commercially available sebocytes share a generally similar gene expression profile with our primary sebocytes, they appear less differentiated and exhibit weaker lipid synthesis capacity. We think there are several factors that may contribute to these differences. The commercially available sebocytes were cryopreserved and thawed prior to use, while our primary sebocytes were freshly isolated and had undergone minimal passaging. Moreover, the commercially available sebocytes were derived from a single 57‐year‐old Caucasian female donor, whereas our primary sebocytes were isolated from multiple donors of varying ages and genders. Since sebocyte function is influenced by variables such as age, sex, ethnicity, lifestyle, and diet, the commercial sebocytes may be less representative overall. Although commercially available sebocytes were useful during the early phases of this study for validating sebocyte identity, their utility for functional characterization appears limited.

Using cultured primary sebocytes, we optimized UV‐A irradiation conditions based on natural sunlight intensity and previous studies [13]. To evaluate whether the UV‐A‐induced cytokine responses were sebocyte‐specific, we conducted parallel experiments using NHDFs. In NHDFs, IL‐1α, IL‐1RA, and IL‐1β levels remained low and largely unchanged, supporting the specificity of these responses in sebocytes. IL‐8 levels were higher in NHDFs but did not respond to UV‐A. These findings support the inflammatory response to UV‐A irradiation being specific in sebocytes. IL‐1α is known to be rapidly released in response to cell damage [14] and exists in a membrane‐bound form capable of initiating inflammation almost immediately [15]. An increase in IL‐1α mRNA upon UV‐A exposure has also been reported in SZ95 cells [16], consistent with our findings. IL‐1RA, a natural antagonist of IL‐1α, competitively binds to the same receptor. While both cytokines increased following UV‐A exposure in our model, inflammation was believed not to be suppressed. The ratio between IL‐1α and IL‐1RA is reported to play a critical role in regulating inflammatory responses [17]. However, the inhibitory effect of IL‐1RA is influenced by multiple factors, including timing, local concentration, and cellular signaling context [15]. Specifically, the secretion and diffusion of IL‐1RA may be delayed or insufficient to counteract the rapid and localized membrane‐bound activity of IL‐1α. Additionally, membrane‐bound IL‐1α is relatively resistant to IL‐1RA inhibition and can induce robust inflammation via direct cell–cell contact. Given that IL‐1α is highly expressed in SG‐related inflammatory diseases and plays a regulatory role in SG inflammation [18], and consistent with these mechanisms, our data show that the absolute release of IL‐1α far exceeded that of IL‐1RA, contributing to sustained inflammation despite their co‐induction. Overall, we propose that UV‐A–induced IL‐1α release from SGs may play a pathogenic role in UV‐A‐induced skin inflammation. In addition, we believe a comparison of sebocytes with keratinocytes in the future study may provide more physiologically relevant insights because of their shared epithelial lineage.

Gene expression analysis revealed a dose‐dependent increase in the expression levels of *DCBLD1*, *ORAI1*, and *BDNF* following UV‐A irradiation. *BDNF*, previously shown to be upregulated in mesenchymal stem cells upon IL‐1α stimulation [19], is a key regulator of immune responses via pathways such as the NF‐κB and JNK pathways [20]. We propose that *BDNF* functions downstream of IL‐1α to mediate SG‐related immune responses. Although *ORAI1* has not been directly linked to SG inflammation, it encodes a calcium channel associated with other Ca^2+^ channels that regulate SG function [21]. Given that sebum secretion is influenced by Ca^2+^ influx [22, 23], *ORAI1* might contribute to sebocyte activity after UV‐A irradiation. Among these genes, *DCBLD1* is the least studied; however, it has been implicated in cell adhesion and is upregulated in certain epithelial cancers [24], suggesting its potential role in UV‐A‐irradiation‐induced skin abnormalities. These findings highlight novel candidates that mediate SG inflammation in response to UV‐A irradiation. However, the interplay among these genes, cytokines, and sebocyte function remains to be fully elucidated, particularly in relation to UV‐A‐irradiation‐induced alterations in sebum composition. As sebum oxidation is considered a key factor in skin inflammation, we investigated whether UV‐A irradiation directly influenced intracellular sebum‐like lipids in sebocytes, potentially acting as a stimulus for inflammatory responses.

It is well established that surface sebum is oxidized by UV‐B light, triggering skin inflammation [4, 25, 26]. However, our study focused on long‐wavelength UV‐A light, which penetrates deep into the dermis and directly influences SGs. In our study, we focused on the detection and quantification of TGs, the major component of human sebum and a lipid species reported to be oxidized by UV‐A irradiation, which can lead to inflammatory responses [4]. We hypothesized that UV‐A irradiation directly affects intracellular sebum‐like lipids within sebocytes, potentially acting as a stimulus for skin inflammation. Furthermore, our study revealed a sex‐dependent response of intracellular TGs to UV‐A irradiation. In females, TGs were more sensitive to UV‐A irradiation, with both saturated and unsaturated TGs undergoing significant changes. Unsaturated fatty acids derived from these TGs are known to trigger skin inflammation and influence organs near the SGs [27, 28]. Given that females exhibit a higher prevalence of inflammatory skin diseases, such as pemphigus [29] and atopic dermatitis [30], we propose that female‐derived SGs were more susceptible to UV‐A‐irradiation‐induced changes, possibly through a mechanism involving sebum oxidation. While this focus on TGs is consistent with previous studies used to assess sebocyte function [31], we acknowledge that the absence of SG‐specific lipids such as squalene, wax esters, and certain free fatty acids limits the ability to fully reflect in vivo sebocyte functionality. The production of these lipids likely requires more complex cues, including 3D architecture and specific cell–cell interactions, which are not present in standard 2D culture systems [32]. In future studies, we will apply specific lipids generated by UV‐A irradiation to a 3D cultured skin model to investigate the precise mechanisms by which these lipids influence the condition of the skin adjacent to SGs. We also aim to investigate other lipid species, particularly long‐chain and short‐chain free fatty acids, to further characterize the lipidomic changes induced by UV‐A.

Consistent with previous reports, we found that the culture supernatant of UV‐A‐irradiated sebocytes induced an increased TEWL ratio in the 3D cultured skin model compared with that in the NT group. This finding, along with our previous results, strongly supports our hypothesis that altered sebum composition and inflammatory cytokines, such as IL‐1α and IL‐1RA, released from UV‐A‐irradiated sebocytes contribute to skin barrier damage [33]. In parallel with the increase in TEWL, *INVL* and *KRT10* expression levels also showed significant increases. Because both *INVL* and *KRT10* are involved in skin barrier formation and maintenance [34], their upregulation further indicated barrier disruption. These findings suggest the possibility that exposure to sebocyte‐derived factors compromised the skin barrier function and initiated a self‐repair response in the 3D cultured skin model. While our in vitro and 3D model data suggest that UV‐A–irradiated sebocytes release factors that impair barrier integrity, whether these factors can diffuse to the epidermis in vivo remains unclear. Therefore, further in vivo studies are needed to validate our findings.

In conclusion, our research demonstrates that UV‐A irradiation influences SG function by altering intracellular sebum‐like lipid composition and triggering inflammation, which may contribute to skin barrier damage. By demonstrating an efficient culture method for primary human sebocytes, we introduced a new approach to SG research, including gene expression profiling that identified several novel markers. These findings not only enhance our understanding of SG responses to environmental stressors but also provide insights into the molecular mechanisms underlying sebum secretion. Moreover, although with limitations, our current culture system is a morphologically stable and functionally responsive monolayer model. The model supports reproducible sebocyte maintenance with active lipid metabolism and offers a practical, scalable platform for drug screening, gene editing, and mechanistic studies. We believe this represents an important step toward translational applications, particularly in acne research and sebum‐targeted therapeutics.

## Author Contributions

W.D. and M.K. conducted the study; M.T. and H.K. provided advice and assisted with specific experiments; A.K. performed the lipid analysis; M.K. and F.F. managed the overall study as co‐corresponding authors. All authors contributed to the study.

## Ethics Statement

This study was conducted at Osaka University and Mandom Corporation. Experiments involving human skin were approved by the Ethics Committee of Osaka University (Certificate Number: yakujin‐2019‐28).

## Conflicts of Interest

M.K., M.T., A.K., and F.F. were employed by Mandom Corporation. The remaining authors declare that the research was conducted in the absence of any commercial or financial relationships that could be construed as a potential conflicts of interest.

## Supporting information


**Figure S1:** jocd70392‐sup‐0001‐FiguresS1‐S2.docx.

**Table S1:** TaqMan gene expression assays with gene symbols and assay IDs.

## Data Availability

Data sharing is not applicable to this article as no new data was created or analyzed in this study.
