# Supplementary material for: UV‐A Radiation Impairs Sebaceous‐Gland‐Related Skin Barrier Function by Inducing Inflammation and Altering Intracellular Sebum‐Like Lipid Composition
Source: J Cosmet Dermatol. 2025 Oct 10;24(10):e70392. doi: 10.1111/jocd.70392 (PMC12512502; doi:10.1111/jocd.70392)
Supplement: Supplementary file 1 — Figure S1: jocd70392‐sup‐0001‐FiguresS1‐S2.docx. [file JOCD-24-e70392-s001.docx]

Supplementary figure 1. RT-qPCR revealed the relative expression of sebaceous gland markers in cultured human primary sebocytes, commercially available sebocytes referred as SK0390 cells, and NHDFs. Data are presented as mean ± SEM.

(a)

(b)

(c)

(d)

(e)

(f)

Supplementary figure 2. Inflammatory cytokines released from NHDFs into the cell culture supernatant. Data are presented as mean ± SEM.

(a)

(b)

(c)

(d)
