# Supplementary material for: UV‐A Radiation Impairs Sebaceous‐Gland‐Related Skin Barrier Function by Inducing Inflammation and Altering Intracellular Sebum‐Like Lipid Composition
Source: J Cosmet Dermatol. 2025 Oct 10;24(10):e70392. doi: 10.1111/jocd.70392 (PMC12512502; doi:10.1111/jocd.70392)
Supplement: Supplementary file 2 — Table S1: TaqMan gene expression assays with gene symbols and assay IDs. [file JOCD-24-e70392-s002.docx]

Supplementary Table S1. TaqMan® Gene Expression Assays with Gene Symbols and Assay IDs

| Gene Symbol | Assay ID |
| --- | --- |
| *DCBLD1* | Hs00920580_m1 |
| *ORAI1* | Hs03046013_m1 |
| *BDNF* | Hs00538277_m1 |
| *MMP1* | Hs00899659_g1 |
| *INVL* | Hs00846307_s1 |
| *KRT10* | Hs00166289_m1 |
| *FLG* | Hs00856927_g1 |
| *LOR* | Hs01894962_s1 |
| *KRT1* | Hs00196158_m1 |
